# Mechanical and Fatigue Properties of Ti-6Al-4V Alloy Fabricated Using Binder Jetting Process and Subjected to Hot Isostatic Pressing

**DOI:** 10.3390/ma17153825

**Published:** 2024-08-02

**Authors:** Jesús Manuel Alegre, Andrés Díaz, Ruben García, Luis Borja Peral, Miriam Lorenzo-Bañuelos, Isidoro Iván Cuesta

**Affiliations:** 1 Escuela Politécnica Superior, University of Burgos, Av de Cantabria s/n, 09006 Burgos, Spain; adportugal@ubu.es (A.D.); mlbanuelos@ubu.es (M.L.-B.);; 2 HIP Innovation Center, Hiperbaric S.L., C. Condado de Treviño, 6, 09001 Burgos, Spain; r.garcia@hiperbaric.com; 3 Materials Science and Metallurgical Engineering Department, Edificio Departamental Este, Campus de Gijón, University of Oviedo, St. Wilfredo Ricart, s/n, 33007 Gijón, Spain; luisborja@uniovi.es

**Keywords:** Ti-6Al-4V, binder jetting, hot isostatic pressing, fatigue properties

## Abstract

Binder jetting 3D printing is an additive manufacturing technique based on the creation of a part through the selective bonding of powder with an adhesive, followed by a sintering process at high temperature to densify the material and produce parts with acceptable properties. Due to the high initial porosity in the material after sintering, which is typically around 5%, post-sintering treatments are often required to increase the material density and enhance the mechanical and fatigue properties of the final component. This paper focuses on the study of the benefits of hot isostatic pressing (HIP) after sintering on the mechanical and fatigue properties of a binder jetting Ti-6Al-4V alloy. Two different HIP processes were considered in this study: one at 920 °C/100 MPa for 4 h, and a second at a higher pressure but lower temperature (HIP-HPLT) at 850 °C/200 MPa for 2 h. The effects of the HIP on the densification, microstructure, mechanical behavior, and fatigue properties were investigated. The results show that the HIP-HPLT process produced a significant increase in the mechanical and fatigue properties of the material compared with the as-sintered parts and even with the conventional HIP process. However, the fatigue and fracture micromechanisms suggest that the oxygen content, which resulted from the decomposition of the binder during the sintering process, played a critical role in the final mechanical properties. Oxygen could reduce the ductility and fatigue life, which deviated from the behavior observed in other additive manufacturing techniques, such as powder bed fusion (PBF).

## 1. Introduction

Binder jetting (BJ) is an additive manufacturing (AM) technique in which a liquid bonding agent is selectively deposited onto powder to create a three-dimensional “green” body, which is then densified through a sintering process. The BJ technique offers several advantages over other additive manufacturing processes. First, binder jetting is compatible with a wide range of powdered materials, including various metals and ceramics. This flexibility enables a broader choice of materials when designing and manufacturing components.

Another key advantage of BJ is that the forming process occurs at room temperature, avoiding issues related to residual stresses and microstructural changes that are typical in laser-based fusion techniques. In selective laser melting, for instance, a heat source is used to melt the powder layers, resulting in significant residual stress in the final part. By contrast, the BJ process requires only relatively low heat during the curing phase (typically temperatures lower than 450 °C). This low-temperature approach reduces the risk of residual stress and thermal distortion. Finally, the sintering process for the BJ green parts takes place at high temperatures, but in a well-controlled and uniform manner. This ensures that residual stresses and thermally induced distortions are minimized, leading to parts with greater dimensional stability.

Regarding the fabrication procedure, the binder jetting technique does not require support structures for complex geometries, resulting in significant time and material savings. This feature facilitates the creation of intricate and detailed designs. Moreover, BJ technology does not require expensive sealed chambers to ensure vacuum or inert atmospheres, reducing equipment costs and enabling larger build volumes. 

Another advantage of the BJ process lies in its high production rate and ability to produce large part volumes; BJ is thus a more cost-effective manufacturing method compared with other additive manufacturing techniques. With build volume capacities up to 2200 × 1200 × 600 mm, BJ can produce sizable components while maintaining a high printing resolution [1]. 

The binder jetting process shares several similarities with metal injection molding (MIM) [2]. In both techniques, metal powder is first mixed with a binder, and then undergoes debonding at a relatively low temperature to produce a “green” part with a low strength and high porosity. This is followed by a sintering process at a temperature below the melting point of the primary constituent to densify the material. Both the MIM and BJ techniques for Ti-6Al-4V yield similar microstructure features and residual porosities after sintering, leading to fatigue properties that are not comparable with those of wrought alloys [3]. Research papers and standards, such as ASTM F2885-11 [4], are more common for MIM than for BJ, and can hence be useful references for assessing BJ technology. 

However, the BJ process for Ti-6Al-4V has several drawbacks compared with other additive manufacturing processes, mainly due to the high porosity of the manufactured part and the coarse microstructure that results from the sintering process. As-printed green parts typically have a low relative density of about 60% of the pore-free density (PFD or absolute density). After the sintering stage, the densification of the material increases up to approximately 95% of the PFD, but this internal porosity is still high and, although the part shows some structural resistance, it is not suitable to withstand demanding stress conditions, such as fatigue. The sintering temperatures needed to achieve optimal PFD values (up to 97%) were studied by Simchi et al. [2], who successfully fabricated high-density Ti-6Al-4V parts with microstructures that were comparable with metal injection-molded titanium parts. Consequently, additional post-processing steps, such as hot isostatic pressing (HIP), are often required for BJ-printed parts to attain acceptable material properties.

Another important drawback of BJ techniques arises from the high reactivity of titanium with some elements at high temperatures, especially with oxygen. Typically, the ink used for the BJ process consists of a water-soluble polymer (binder) that is dissolved in mainly water. This water-based agglomerant is almost completely removed from the initial component during the de-binding stage prior to sintering. However, any residual binder constituents are decomposed into their elemental constituents during the sintering cycle. The amount of the residual binder constituents in the MIM components is controlled through the chemical requirements in ASTM F2885-11 [4].

Oxygen is one of the most critical impurities that affects the mechanical properties of titanium alloys [5,6,7,8]. An increase in the oxygen content leads to a higher tensile strength but significantly reduces the material ductility. Yan et al. [5,8] investigated the effect of oxygen on the tensile and fatigue properties, as well as the microstructural changes induced by a high oxygen content. They examined several MIM as-sintered Ti-6Al-4V alloys that contained 0.25%, 0.33%, and 0.49% oxygen. Although the overall microstructures appeared similar, detailed studies revealed distinct microstructural features caused by a high oxygen content, specifically the presence of acicular α precipitates in the β phase [8]. According to the ASTM F2885-11 standard [4], the oxygen content in titanium alloys should be limited to a maximum of 0.35 wt.% because elongation decreases rapidly when oxygen levels exceed this threshold.

In addition to oxygen, other impurities, such as carbon (C) and nitrogen (N), which can be introduced from polymeric binders during the debonding and sintering stages, also impact the mechanical properties of binder jetting and MIM Ti-6Al-4V alloys. These impurities can further reduce the ductility and affect the fatigue life of the resulting components.

Hot isostatic pressing (HIP) is currently one the best processes to improve the performance of additively manufactured parts. This is an effective technique to virtually eliminate the internal porosity and densify the material, reaching values up to 99.9% of the PFD. This is a thermomechanical post-treatment in which a component is subjected to a combination of high pressure and high temperature for a holding time in an inert-gas-pressurized environment. The main objective is to reduce the porosity and increase the material density. This technology was initially developed during the 1950s as a means of removing the porosity in hard metals in the nuclear sector [9]. Nowadays, its use is mainly focused on the densification of high-performance castings and the consolidation of metal powders, including additive manufacturing techniques in many industries, such as the aerospace, biomedical, oil and gas, power generation, and automotive industries. The increased fatigue resistance of Ti alloys after HIP can not only be attributed to porosity reduction but also to microstructure changes. For instance, Guo et al. [10] demonstrated the beneficial role of the lamella globularization leading to equiaxed microstructures. On the other hand, finer α lamellae can also improve the resistance to microcrack propagation [11].

Numerous studies examined the benefits of HIP treatment on the defects, microstructure, and mechanical properties of additively manufactured Ti-6Al-4V alloys produced using powder bed fusion (PBF), which is also termed in the literature as selective laser melting (SLM) [12,13,14,15,16,17,18,19,20,21].

A typical HIP cycle used for Ti-6Al-4V alloys manufactured by PBF involves heating to a temperature in the range of 900–920 °C (below the β-transus temperature) under a pressure of 100–120 MPa for a holding time between 2 and 4 h in an argon atmosphere, which is typically followed by furnace cooling. 

Some researchers experimented with higher temperatures (950–1000 °C) and observed that certain material properties decrease with increased heat treatment temperatures due to microstructural coarsening. Cai et al. [22] found that for an HIP process at 120 MPa, low temperatures could produce weak metallurgical bonding, whereas temperatures above the β-transus temperature reduced the tensile properties due to the transformation to coarser lamellar microstructures. Conversely, other studies demonstrated the benefits of HIP treatments at lower temperatures (820–850 °C) but with higher pressures (up to 200 MPa), combined with rapid cooling to limit these microstructural coarsening effects and improve the fatigue life of the PBF components [20,21]. 

This study explored the effect of HIP post-treatment on Ti-6Al-4V alloy produced by a binder jetting process. Two different HIP cycles were analyzed in this study: one conventional cycle at 920 °C/100 MPa for 4 h, and a second at a lower temperature but higher pressure (HIP-HPLT) at 850 °C/200 MPa for 2 h. The impacts of the HIP on the material densification, microstructure, and tensile and fatigue properties were examined.

## 2. Materials and Methods

The material used in this research was a Ti-6Al-4V alloy. Specimens were fabricated using high-precision binder jetting technology. The manufacturing process involved building specimens layer by layer by applying a binder to each layer of metal powder. The binder was a water-soluble polymer, and the ink used was water based. Printing was performed at room temperature in a build box that did not require a protective atmosphere. Once the build was complete, parts were removed from the powder bed and sintered to produce an appropriate strength and densification. The average surface finish was 7 µm Ra after sintering, which improved to 4 µm Ra after sand blasting. The layer thickness used in this process was 42 µm.

The sintering process consisted of two stages. First, the binding agent was removed by heating the parts in an argon environment for three hours at 295 °C, which is a process known as de-binding. Second, the Ti-6Al-4V was sintered by heating up to 1350 °C for three hours, followed by furnace cooling. A shrinkage close to 20% during sintering was produced. An oxygen level of about 0.35% was estimated from the obtained experimental tensile tests (elongation, YS, and UTS) [5] and also confirmed with concentration measurements using a LECO 736 analyzer at Azkerlan (LECO Corporation, St. Joseph, MI, USA) laboratories. 

The specimens were divided into three different batches. The first batch was used for studying the “as-sintered” condition, while the second and third batches were treated with different hot isostatic pressing (HIP) processes, with both performed in an argon environment at two different sub-β-transus temperatures.

The HIP processes were applied at the HIP Innovation Center of Hiperbaric (Spain). One set of specimens was subjected to an HIP cycle at 920 °C/100 MPa for 4 h, followed by furnace cooling. Another set, which was defined as a low-temperature but high-pressure cycle (HIP-LTHP), underwent a treatment at 850 °C/200 MPa for 2 h, followed by forced cooling to minimize the holding time at high temperatures.

All specimens were machined and finished to their final geometry after the sintering or HIP stages. Monotonic tensile tests were carried out using cylindrical specimens according to the ASTM E8M standard [23] using smaller-sized specimens proportional to the standardized size, with a diameter of 6 mm. A linear extensometer model EPSILON with a 20 mm gauge length was used, and tests were conducted on an MTS 810 servo-hydraulic testing machine (MTS System, Eden Prairie, MN, USA).

**Figure 1:**
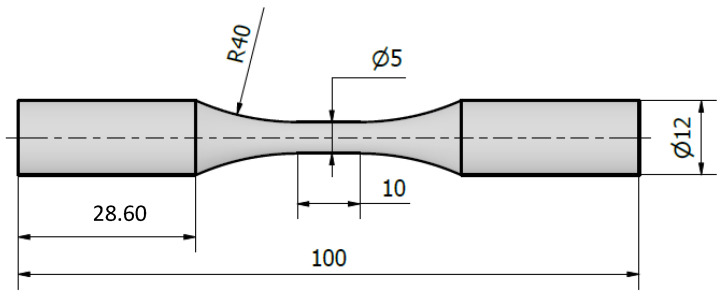
Fatigue specimen geometry (dimensions in mm).

For the fatigue analysis, the specimens shown in Figure 1 were used. The surface roughness was checked after machining, where the Ra values ranged from 0.61 to 0.88 µm for all samples. The fatigue behavior was characterized through constant amplitude tests under tension–tension (R = 0.1) at a frequency of 15 Hz and at a temperature of 20 °C. Fatigue tests were carried out on a servo-hydraulic test machine MTS-810.

For the metallographic analysis, the microstructures were revealed using a Kroll’s reagent (consisting of 100 mL of distilled water, 2 mL of hydrofluoric acid [HF], and 4 mL of nitric acid [HNO_3_]) to etch the samples that were previously ground and polished.

Scanning electron microscopy (SEM) was performed using a high-resolution field-emission SEM (JEOL JSM-6460LV, JEOL, Akishima, Janpan) operated at 20 kV at the R&D Center at the University of Burgos, Spain. The optical microscope used for the metallographic analysis was a Leica DMi8 M inverted microscope (Leica Microsystems, Wetzlar, Germany).

## 3. Results and Discussion

### 3.1. Microstructure

**Figure 2:**
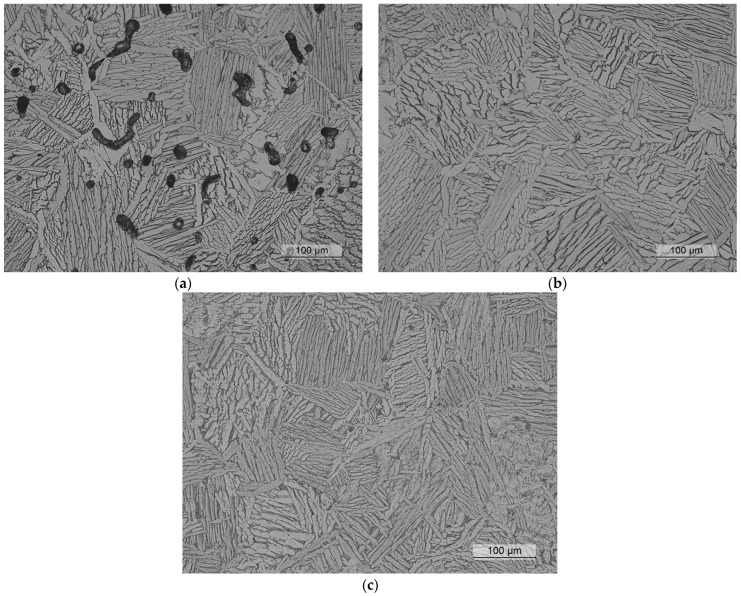
Microstructure of the three conditions analyzed in this study: (**a**) as-sintered, (**b**) HIP at 920 °C/100 MPa for 4 h, and (**c**) HIP at 850 °C/200 bar for 2 h.

Figure 2 shows the microstructure of the different conditions analyzed in this study. In the as-received condition, a uniformly distributed porosity was observed, with irregular aspect ratios. Some elongated pores were present, as shown in Figure 2a. The matrix exhibited a fully lamellar microstructure with distinct regions of α and β phases. These characteristics are similar to those reported for metal injection molding (MIM) Ti-6Al-4V alloys [2]. 

**Figure 3:**
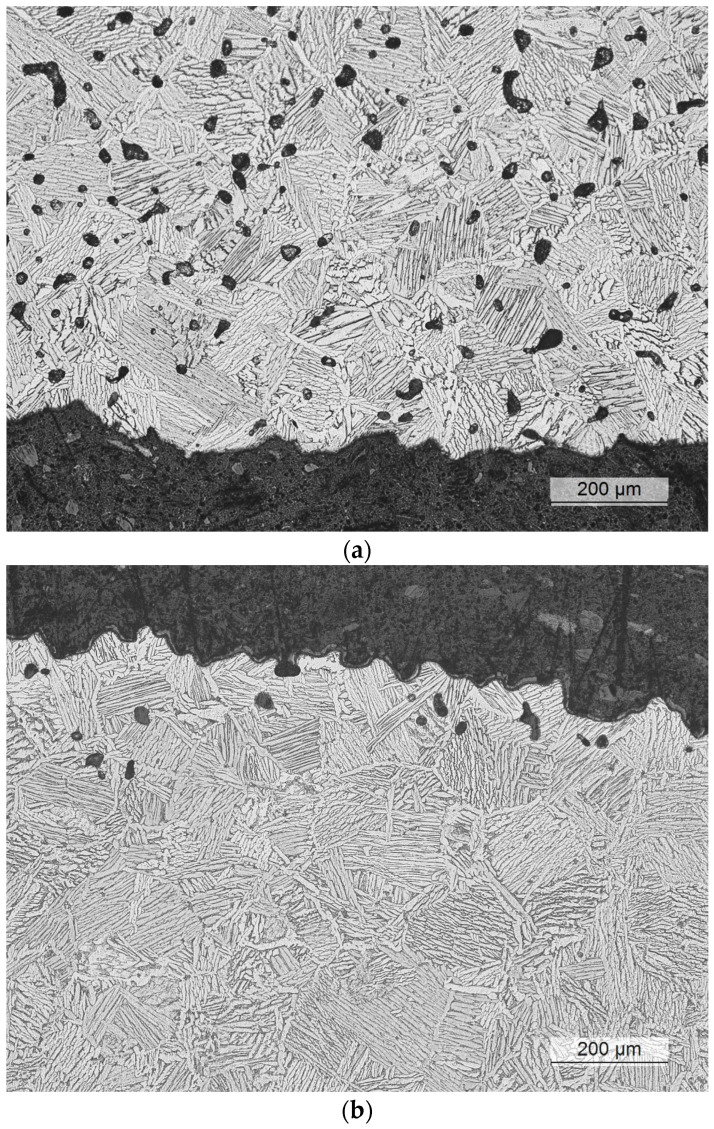
Microstructure of BJ at the surface of the as-sintered (**a**) and HIP at 850 °C/200 bar for 2 h (**b**). Dark areas correspond to the polymeric mounting material.

After the HIP process, nearly all the internal voids were eliminated, as observed in Figure 2. However, HIP was only effective for sealing internal voids, and some isolated voids could still be found near the surface. These were not removed due to the prior elongated voids that were connected to the external surface of the part, as shown in Figure 3. During the HIP process, the pressurized gas entered into these elongated and interconnected voids, preventing them from being subjected to the hydrostatic compression stress required to seal them. This phenomenon occurred in both the HIP treatments and could impact the fatigue behavior of components used in as-built surface conditions.

The objective of this study was to analyze the effect of the HIP post-treatment on the densification and microstructure of the material. To this end, the specimens were machined after the sintering and HIP to remove the outer layer where these surface voids were likely to be present. Future research should focus on the effect of HIP on the fatigue life of components in as-built surface conditions to better understand how subsurface unsealed voids might impact fatigue crack initiation and reduce the effectiveness of HIP treatments.

Micrographs for the as-sintered and for both HIP conditions were analyzed using the post-processing capabilities of the Leica Application Suite software (version 4.12.0) and its Grain Expert module. Measurements were carried out to determine the α-lath thickness and the α-colony size. In addition, the porosity was measured for the as-sintered condition. The determination of a specific value for the size of the α colonies was not straightforward since the boundaries were not clearly defined in many groups of α laths. Advanced image processing strategies are required, e.g., gradient orientation assessment or Watershed Transform techniques [24], but they were out of the scope of this work. For the three analyzed conditions, 40 α colonies were measured in a 10× image to find an average equivalent diameter around 110 µm without a clear influence of the HIP post-process.

**Figure 4:**
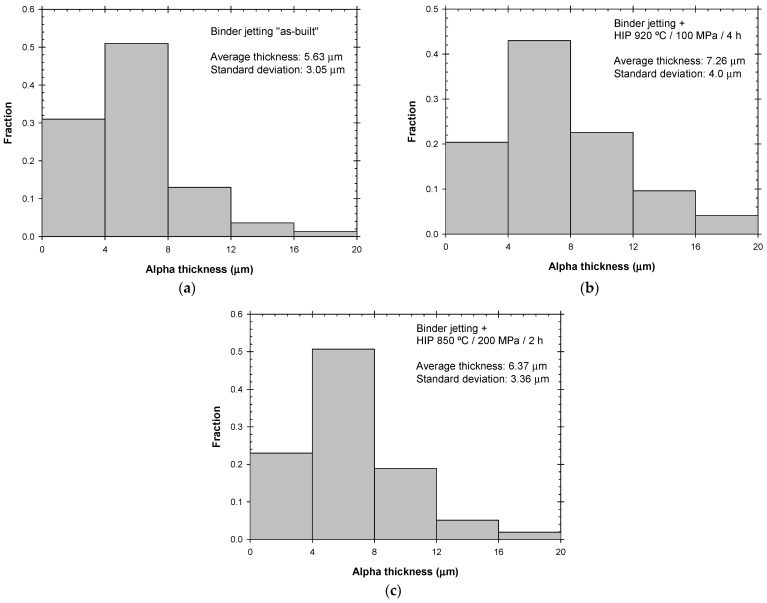
Histograms showing the α-thickness distribution for (**a**) as-sintered, (**b**) HIP at 920 °C/100 MPa for 4 h, and (**c**) HIP at 850 °C/200 MPa for 2 h.

Figure 4 shows the distribution of the α-phase thickness, as measured using a micrograph at 10× magnification over a region of approximately 1 mm^2^. Unlike the thin acicular α microstructure typically observed in selective laser melting (approximately 1 µm [21]), binder jetting produces coarser α laths. The average thickness for the as-sintered condition was 5.63 µm, while the HIP post-treatment slightly increased this value to 6.37 µm for the low-temperature HIP and to 7.26 µm for the conventional HIP process. As expected, the higher temperatures and longer holding times resulted in coarser microstructures. Figure 4c illustrates how the fraction of α grains with a thickness below 4 µm was reduced by HIP, while the fraction of those with a thickness greater than 8 µm increased, which demonstrated the coarsening effect of higher temperatures and longer holding times.

### 3.2. Monotonic Tensile Properties

**Figure 5:**
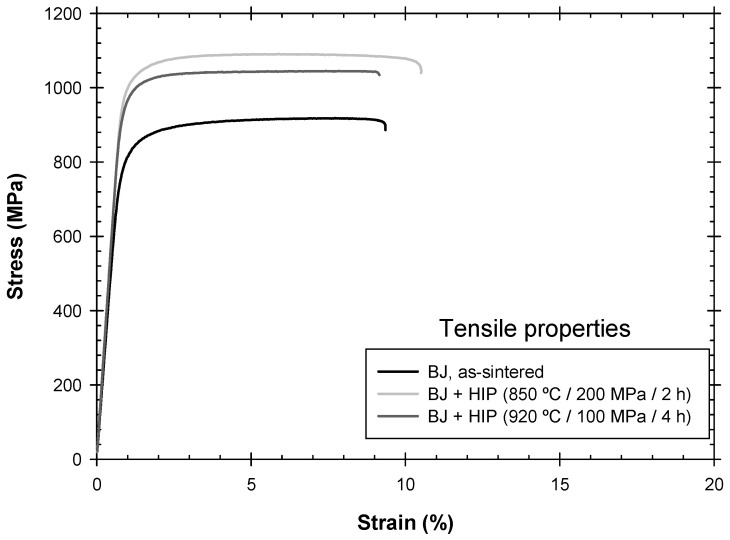
Representative tensile test curves for BJ as-sintered, BJ + HIP, and BJ + HPLT of Ti-6Al-4V.

Experimental tensile test curves for the three analyzed conditions are presented in Figure 5, and the key tensile properties are summarized in Table 1. For comparison, the results for a reference wrought-processed material and a material manufactured by PBF are also included [21]. 

A notable observation from these results is the lower elongation capacity of the binder jetting material, which was approximately 9%, except for the material processed by HIP-HPLT, where the elongation slightly increased to 10.5%. These values were significantly lower than the elongation capacity of the reference wrought material (19.5%). In addition, this elongation value of the BJ condition, even after HIP-HPLT, was also lower than in the same material manufactured by PBF and subjected to the same HIP-HPLT treatment, which generally achieved an elongation in the range of 11–13%, as reported by the same authors in previous studies.

Some interesting conclusions could be drawn regarding the tensile properties of the materials under different conditions. The reference wrought material was characterized by equiaxed primary α grains with an average size of 5.92 µm and β-phase grain boundaries. It exhibited a moderate yield strength of 894 MPa and good ductility with an elongation of 19.5%, along with excellent fatigue behavior, as reported in a previous work by the authors [21].

The as-built PBF material was characterized by an acicular microstructure with martensitic α-phase laths that were approximately 1 µm thick. This material had a higher yield strength (1090 MPa) but a lower ductility (11.2%). Its fatigue properties were generally poor due to large internal defects, like a lack of fusion or entrapped gas pores.

The PBF material subjected to the HIP-HPLT treatment demonstrated a good combination of strength, with a yield strength of around 945 MPa, a moderate deformation capacity of approximately 13.2%, and good fatigue behavior close to that of the reference material. The HIP process nearly sealed the internal defects generated by the PBF process while maintaining a fine-grained microstructure.

In the binder jetting Ti-6Al-4V alloy analyzed in this study, the as-sintered condition showed a poor tensile strength of 790 MPa and a reduced deformation capacity of about 9.25%. These poor mechanical properties could be attributed to the high level of porosity after sintering, which could reach up to 5% (as shown in Figure 2 and Figure 3). The fatigue properties also showed similar deficiencies, as discussed in detail in the following section.

The HIP process applied to the binder jetting specimens effectively sealed the internal pores and channels formed during the sintering stage. HIP provided excellent densification, except for the voids and defects connected to the surface, as mentioned earlier. The tensile properties obtained after the HIP treatment demonstrated an appropriate strength value that was comparable with that of the PBF + HIP process, but the deformation capacity was lower, not exceeding 10%. A slight improvement in the performance was observed with HIP at a low temperature and high pressure.

**Figure 6:**
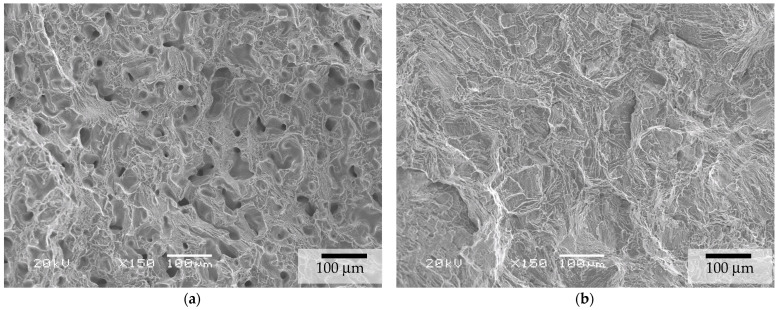
Fracture aspect of tensile tests for (**a**) BJ as-sintered and (**b**) BJ + HIP HPLT.

**Figure 7:**
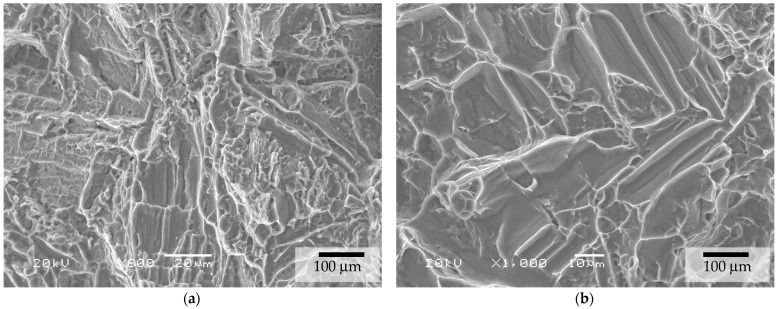
Details at ×800 (**a**) and ×1000 (**b**) magnification of the fracture aspect of tensile tests for the BJ + HIP HPLT conditions.

For the as-sintered and HIP BJ samples, the fracture surface had the overall appearance shown in Figure 6. Detailed micrographs revealed that fractures occurred due to intergranular propagation mechanisms at the β-phase interface between the α laths. The fracture surface detail of the HIP samples is presented in Figure 7.

### 3.3. Porosity Measurements

The relative densities of the samples were measured using the Archimedes principle of buoyancy, in accordance with ASTM B311 [25], with the theoretical density of Ti-6Al-4V set at 4.43 g/cm^3^. The test was performed using an AJH-420 CEN electronic balance with a precision of 0.001 g, along with a density measurement kit.

After sintering, the measured relative density was 96.0%. After the hot isostatic pressing (HIP) process, the relative density increased to 99.0%, indicating that the material was not completely dense, which was likely due to the open porosity in the as-sintered samples. After the machining, the relative densities of both the as-sintered and HIP specimens was remeasured, which yielded values of 96.0% and 99.9%, respectively.

Additionally, the number of pores in the as-sintered condition was quantified from a 20× micrograph over an area of approximately 0.53 mm^2^. About 87 pores were measured, with an average equivalent diameter of 18 µm and a 2D porosity—which is defined as the total area of pores divided by the total area analyzed—of 4.1%. Although the volumetric void fraction could be estimated from this 2D analysis using Delesse’s principle [26], this approach typically underestimates the 3D porosity [27]. Stereographic theory should be applied for a more accurate correlation between 2D and 3D porosity values.

### 3.4. Fatigue Properties and Microstructural Aspects

**Figure 8:**
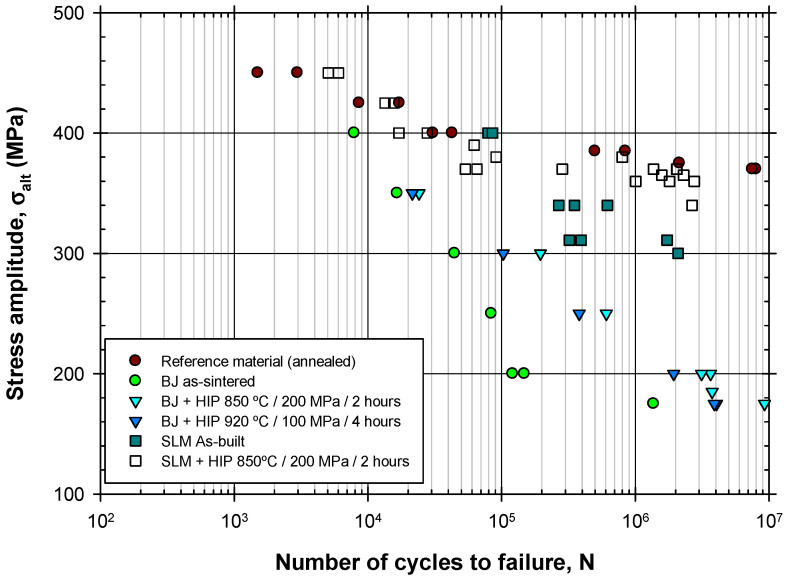
Fatigue test results for the different conditions analyzed, along with the behavior of the base material and the same alloy manufactured by PBF.

Constant amplitude stress levels were applied with a stress ratio (R = 0.1) at a frequency of 15 Hz. The stress amplitude against the number of cycles until failure, including the complete set of results for all the different batches, is plotted in Figure 8. For comparison, the results for a reference wrought material and a material manufactured by PBF are also included [21].

As can be observed, the fatigue behavior of the BJ material exhibited a poor fatigue resistance, with fatigue limit values below 200 MPa, which are significantly lower than those of the reference wrought material. Internal fatigue initiation sites were not common. For the BJ + HIP conditions, the fatigue limit was still below 200 MPa. Although an improvement was observed compared with the BJ as-built material, its fatigue limit was much lower than that of the material manufactured by PBF.

The poor fatigue resistance of BJ material could be attributed to the high internal porosity after sintering, the thickness of the α laths, and the oxygen content that promoted the formation of oxygen-enriched precipitates. These factors increased the local stress concentrations, and thus, reduced the fatigue performance. 

**Figure 9:**
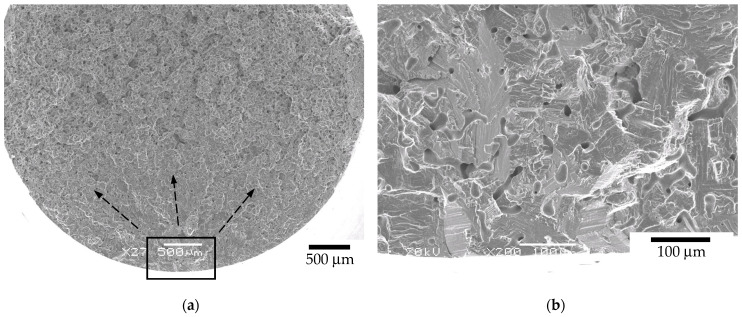
Fatigue initiation site (**a**) and magnified detail (**b**) for BJ as-sintered conditions.

**Figure 10:**
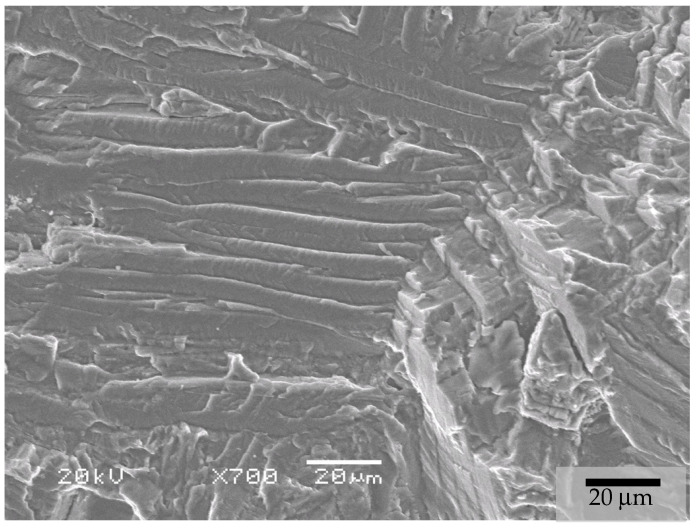
Crack initiation site that resulted from facet formation in binder jetting (BJ) as-sintered conditions.

The crack initiation mechanism in the as-sintered condition exhibited a characteristic pattern, as shown in Figure 9 and Figure 10. Multiple flat facets were observed at the initiation site, along with numerous voids that resulted from the sintering stage. This combination of defects promoted multiple internal stress concentrators, which led to poor fatigue behavior. Crack initiation in α/β titanium alloys is well documented [28,29,30,31,32] and is consistently associated with the formation of quasi-cleavage facets [1]. In this case, the sizes and shapes of these facets corresponded to the α laths and exhibited a quasi-cleavage appearance, as can be observed in Figure 10.

For the material subjected to HIP, the formation of facets with a brittle fracture appearance, which originated in the α phase, was also observed. Although the fatigue behavior of the HIP-treated material was better than that of the as-sintered BJ condition, as presented in Figure 8, it was still significantly lower than the fatigue performance of similar materials produced by other additive manufacturing processes, such as PBF [21], even in their initial as-built conditions, i.e., without the HIP treatment. Fatigue initiation in the HIP-treated material also occurred from facet formation in the α+β phase, which is a mechanism that aligns with those previously observed in PBF-produced materials.

**Figure 11:**
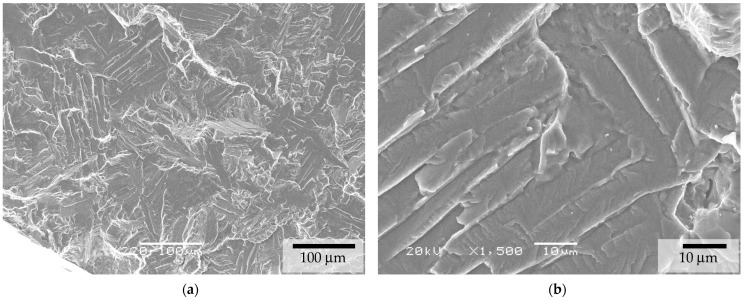
Fatigue initiation site for the BJ + HIP-HPLT condition (**a**) and magnified detail at ×1500 of the facets (**b**).

**Figure 12:**
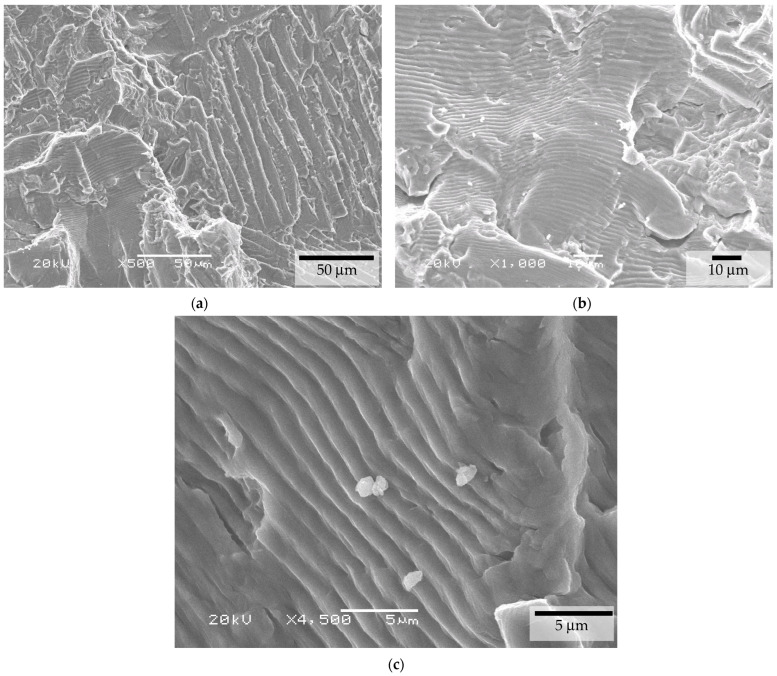
Fatigue propagation site for the BJ + HIP-HPLT condition (**a**), showing facets mixed with the typical fatigue crack growth striations at ×1000 magnification (**b**) and details at ×4500 magnification of the O-rich Ti dispersoids (**c**).

In some regions, fatigue crack propagation occurs between the α-phase laths. Instead of facets perpendicular to the laths, propagation occurred between them, which generated the typical fatigue striations. The poor fatigue behavior could also be attributed to the residual oxygen content in the BJ material, which, in this study, reached 0.35%. Figure 11 shows a micrograph of the crack initiation site for these BJ + HIP conditions, which revealed the presence of particles on the fracture surface that were identified as oxygen-enriched titanium dispersoids, which were also present in the fatigue crack regions (Figure 12c). The composition of these particles was determined by SEM-EDS techniques. This is also common in materials produced by MIM when the oxygen content exceeds certain levels, leading to reduced ductility and drastically lowering the fatigue properties.

Due to the local increase in hardness and decrease in ductility, oxygen-enriched regions in Ti-6Al-4V act as initiation sites for fatigue cracks. The hardening effect is expected to lead to higher stress at grain boundaries and other discontinuities, promoting crack initiation. Therefore, an increase in oxygen content leads to a reduction in fatigue life for Ti-6Al-4V, contributing to both an earlier crack initiation and to faster propagation rates due to reduced ductility and an enhanced stress concentration.

Figure 12 shows the appearance of the crack propagation for the BJ material subjected to the HIP treatment. The crack propagation occurred from the formation of facets in the α+β phase. The propagation shows the existence of these facets mixed with the typical fatigue crack growth striations, which resulted from the propagation through slab interfaces when the orientation of the slabs relative to the load axis favored this mode of propagation. Also, the O-rich Ti dispersoids are presented in Figure 12c. 

## 4. Conclusions

The effects of two different HIP post-treatments for a Ti-6Al-4V alloy fabricated by BJ on the material densification and mechanical and fatigue behaviors were studied. The following conclusions could be drawn.

The tensile and fatigue performance of the as-built condition (without post-treatment after sintering) was significantly poor in terms of the strength, deformation capacity, and fatigue life. This was primarily due to the high residual porosity after sintering (around 4%) and residual oxygen from binder remnants after debinding, which formed oxygen-enriched dispersed titanium particles in the material matrix.

After the HIP treatment, the tensile strength improved to levels comparable with Ti-6Al-4V produced by other methods, such as PBF. However, the deformation capacity remained limited, which was likely due to the oxygen content in the sample, which was around 0.35%. The fatigue life also improved compared with the as-built condition, which reflected better densification. However, these improvements were still lower than those observed in PBF samples subjected to the same HIP treatment and the reference wrought material.

To utilize the Ti-6Al-4V manufactured by binder jetting (BJ) in components intended for high-load conditions, HIP post-treatment is required to densify the material. Additionally, binder jetting processes or alternative binders need to be developed in order to limit the oxygen content after de-binding to values below 0.3%, as indicated for similar techniques, like MIM.

This study demonstrated that BJ-fabricated Ti-6Al-4V had notable challenges related to the high residual porosity and oxygen content, which impacted its mechanical and fatigue properties. While the HIP improved the material strength and densification, the deformation capacity remained limited, which was likely due to the residual oxygen from the binder process. This research indicates that further improvements in the BJ process, such as reducing the binder-related oxygen content, are essential to achieve mechanical and fatigue properties comparable with other additive manufacturing methods, like selective laser melting. Strategies to reduce the oxygen content to below 0.3% and ensure complete densification are crucial for the successful application of BJ-fabricated Ti-6Al-4V for demanding cyclic loading conditions.

## Figures and Tables

**Table 1:** Mechanical properties of Ti-6Al-4V for different BJ and PBF [21] conditions.

Material	Offset Yield Strength, Rp_0.2_ (MPa)	Tensile Strength, Rm (MPa)	Elongation at Break (%)	Vickers Hardness
Binder jetting as-sintered	790	912	9.35	336
BJ + HIP 850 °C/200 MPa/2 h	1002	1089	10.58	369
BJ + HIP 920 °C/100 MPa/4 h	974	1043	9.25	364
PBF as-built	1090	1297	11.2	362
PBF + HIP 850 °C/200 MPa/2 h	945	1055	13.2	332
Wrought processed material	894	966	19.5	320

## Data Availability

The original contributions presented in the study are included in the article, further inquiries can be directed to the corresponding author.
